# Comprehensive single-cell and bulk RNA-seq analyses reveal a novel CD8^+^ T cell-associated prognostic signature in ovarian cancer

**DOI:** 10.18632/aging.205966

**Published:** 2024-06-25

**Authors:** Yi-Ling Han, Zhou Fang, Zhi-Jie Gao, Wen-Ge Li, Jing Yang

**Affiliations:** 1 Center for Reproductive Medicine, Renmin Hospital of Wuhan University, Wuhan, China; 2 Department of Breast and Thyroid Surgery, Renmin Hospital of Wuhan University, Wuhan, Hubei, China; 3 Department of Oncology, Shanghai Artemed Hospital, Shanghai, China

**Keywords:** ovarian carcinoma, CD8+ T cells, scRNA-seq

## Abstract

CD8^+^ T cells play pivotal roles in combating intracellular pathogens and eliminating malignant cells in cancer. However, the prognostic role of CD8^+^ T cells in ovarian carcinoma is insufficiently exploited. Herein, through univariate Cox regression along with least absolute shrinkage and selection operator (LASSO) regression analyses, we developed a novel prognostic model based on CD8^+^ T cell markers identified by single-cell sequencing (scRNA-seq) analyses. Patient grouping by the median risk score reveals an excellent prognostic efficacy of this model in both training and validation cohorts. Of note, patients classified as low-risk group exhibit a dramatically improved prognosis. In addition, higher enrichment level of immune-related pathways and increased infiltration level of multiple immune cells are found in patients with lower risk score. Importantly, low-risk patients also exhibited higher response rate to immunotherapies. Summarily, this developed CD8^+^ T cell-associated prognostic model serves as an excellent predictor for clinical outcomes and aids in guiding therapeutic strategy choices for ovarian cancer patients.

## INTRODUCTION

Ovarian cancer has the worst prognosis and highest mortality rate among gynecological malignancies worldwide, and is usually diagnosed in advanced stages [1]. Historically, ovarian cancer was consistently treated without considering the distinct biological features among patients, and this trend continues to prevail. In recent years, with increasing recognition of tumor heterogeneity, accumulating studies have emphasized the importance of adopting a standardized and molecular marker-based approach for accurately categorizing ovarian cancers [2]. However, the lack of current powerfully efficient biomarkers and prognostic signatures for ovarian cancer is primarily responsible for the poor prognosis. In summary, the limited treatment efficacy and unfavorable outcomes of ovarian cancer patients make it particularly urgent to develop a novel prognostic signature and guide individual treatment plans for ovarian cancer.

The tumor microenvironment (TME) acts as a complex ecosystem consisting of malignant cells, diverse infiltrating immune cells (lymphocytes and myeloid cells), and stromal cells intertwined with non-cellular components. Tumor-infiltrating T cells are critical players in the TME and influence essential clinical properties, such as cancer immunotherapy responses, with a particular significance for CD8^+^ T cells. The association between the infiltration of T cells in cancer lesions and improved patient prognosis has been widely recognized in various human malignancies for a significant period of time. For example, in human melanoma, the presence of abundant T cell infiltrates has been reported to be linked with improved overall survival (OS) for over two decades [3]. Subsequent studies have revealed that the extent of intra-tumoral T cell infiltrates serves as an independent positive prognostic indicator in ovarian cancer, colorectal cancer and numerous other malignancies [4–6]. These findings highlight the significance of T cell infiltration as a reliable marker for improved patient outcomes across multiple cancer types. As universally acknowledged, the simplest distinction among T cells is the CD4^+^ and CD8^+^ T cell compartments. The CD8^+^ T cell subset has been extensively studied and shown to have a crucial role in tumor control. Accumulating prognostic analyses consistently demonstrate a positive association between the number of pretreatment intra-tumoral CD8^+^ T cells and the response to PD-1 blockade [7]. Furthermore, clinical studies have also demonstrated the effectiveness of CD8^+^ T cell-enriched cell products in melanoma treatment [8]. These findings emphasize the significant contribution of CD8^+^ T cells in tumor immunity and therapy. Nevertheless, a robust prognostic model based on the specific gene markers of CD8^+^ T cells is still absent in ovarian cancer.

The technological advancements in scRNA-seq have brought about a revolution in our ability to profile transcriptomes in thousands of individual cells, which has allowed for the comprehensive and unbiased analysis of the intricate cellular components within tumors. Recent work based on scRNA-seq analyses of malignant and nonmalignant ovarian tissues was successfully applied to delineate a cellular landscape of ovarian cancer with a focus on the heterogeneity of TME [9]. Here, we selectively reanalyzed the transcriptome data of above seven malignant ovarian tissues and identified distinct subpopulations of lymphocytes. The specific gene markers of CD8^+^ T cells in ovarian cancer were interrogated by Wilcoxon rank-sum test algorithm at single-cell resolution. Subsequently, we established a prognostic signature by performing univariate Cox regression and LASSO regression analyses within The Cancer Genome Atlas (TCGA) ovarian cancer training cohort. The potential of prognostic model was further validated in additional ovarian cancer cohorts. Additionally, we investigated differential pathway activities, mutational and immune statuses between the high- and low-risk groups stratified by the median of the risk score. We also made predictions regarding the immunotherapy response and sensitivity to multiple potential anti-cancer drugs based on our risk classification. Finally, a nomogram was developed to offer guidance for the diagnosis and treatment of ovarian cancer. In summary, our study introduces a novel and robust prognostic signature associated with CD8^+^ T cells in ovarian cancer, which can be instrumental in guiding the diagnosis and treatment of ovarian cancer.

## MATERIALS AND METHODS

### Single-cell transcriptome analysis

We obtained scRNA-seq data from seven ovarian tumors from the Gene Expression Omnibus (GEO) database (GSE184880) for this investigation [9]. The Seurat package [10] (v4.1.1) in R (v4.1.3) was used to perform unsupervised clustering of individual cells using the read count matrix as input. Rigorous quality control measures were applied, focusing on the number of detected genes and the percentage of mitochondrial gene counts per cell. Cells with fewer than 200 detected genes and those with more than 20% mitochondrial gene counts were systematically excluded from the analysis. We also omitted genes detected in fewer than 3 cells to reduce potential extraneous signals. To address batch effects, we employed the Harmony algorithm to integrate multi-sample data and correct any inconsistencies. Following the Seurat-guided tutorial, we performed dimension reduction clustering and differential expression analysis. Principal component analysis (PCA) and uniform manifold approximation and projection (UMAP) dimension reduction were conducted based on the top 20 principal components. Cell cluster annotations were determined using canonical gene markers.

### Collection of public datasets

The RNA-sequencing expression matrix and clinical information of ovarian cancer samples and para-cancerous tissues were downloaded from TCGA database, available at UCSC Xena (https://xena.ucsc.edu/). Two additional independent datasets (GSE30161 and GSE63885) were obtained from the GEO database (https://www.ncbi.nlm.nih.gov/geo/) [11, 12]. The somatic mutation data were retrieved from the Genomic Data Commons (GDC, https://portal.gdc.cancer.gov/). The somatic mutation data were then analyzed and sorted in the form of Mutation Annotation Format (MAF), followed by using the R package maftools [13] to calculate tumor mutation burden (TMB).

### Construction and validation of a CD8^+^ T cell-related prognostic signature

Firstly, we collected 1,020 genes associated with CD8^+^ T cells through single-cell transcriptome analysis. We then analyzed the RNA expression matrices of breast cancer samples in the TCGA, GSE30161, and GSE63885 datasets to identify co-expressed genes. To develop a prognostic signature using CD8^+^ T cell-related genes, we performed univariate Cox regression and LASSO regression analyses. In the end, we identified 17 genes: *SLFN5*, *CD40LG*, *EMB*, *ISG20*, *CD226*, *CCR7*, *S1PR4*, *RNF167*, *MLLT3*, *PLEKHF1*, *RNPEPL1*, *STK39*, *FXYD5*, *BTN3A3*, *WNK1*, and *SUSD3*. Based on these genes, we constructed a CD8^+^ T cell-related prognostic model.

To classify ovarian cancer patients, we calculated the risk score for each patient in the training set using the following formula:


Risk score=∑ni=∑(Coefi∗xi)

Next, we divided the ovarian cancer patients into high-risk and low-risk groups based on the median of the risk score. We used the R package survivalROC [14] to estimate the predictive sensitivity of the risk score. Finally, we evaluated the effectiveness of the model in the validation set using the same coefficients and cutoff values as the training set.

### Biological functional analysis between high/low-risk group patients

The Deseq2 [15] R package was used to analyze differentially expressed genes (DEGs). DEGs were identified using a cutoff of an adjusted p-value of < 0.05 and a |Log2 fold change| > 1. Gene set enrichment analysis (GSEA) was conducted with the clusterProfiler R package [16]. Gene sets with false discovery rate (FDR)-corrected p-values < 0.05 were considered significant using Fisher’s exact test. Gene set variation analysis (GSVA) was performed using the R package GSVA [17]. Gene signatures representing recurrent cancer cell states were obtained from a previous study [18].

### Tumor immune microenvironment in ovarian cancer patients

We used the “estimate” algorithm to calculate immune scores for ovarian cancer samples [19]. To analyze the infiltration of immune cells, we used TIMER2.0, an algorithm that efficiently predicts immune cell infiltration based on bulk tumor gene expression data (http://timer.cistrome.org/). Using CIBERSORT, we quantified the relative abundance of 22 immune cells for each sample. Additionally, we gathered a collection of tumor immunomodulators from literature and calculated the correlation between the risk score and these modulators.

### Predicting drug responses and immunotherapy sensitivity

The tumor immune exclusion score was obtained from TIDE (http://tide.dfci.harvard.edu/). The Immunophenoscore (IPS) was calculated using The Cancer Immunome Atlas (https://tcia.at/). To evaluate the predictive ability of the risk score for chemotherapeutic agents, we used the R package oncoPredict [20] to calculate patients’ half maximal inhibitory concentration (IC50) for various common chemotherapeutic agents. The Wilcoxon rank test was then employed to compare the difference in IC50 between the high-risk and low-risk groups. Additionally, we utilized the IMvigor210 dataset (specifically for uroepithelial carcinoma) to further validate the predictive value of the aforementioned immunotherapy responses.

### Univariate and multivariable Cox regression

We performed univariate Cox regression analysis to investigate the correlation between gene expression and OS in patients with ovarian cancer. In the same group, we utilized multivariate Cox regression to identify independent risk factors. Genes and factors that displayed a FDR < 0.05 were deemed statistically significant in terms of patient survival. The outcomes of both univariate and multivariate Cox regression analyses were obtained and visually represented using the R package forestplot.

### Establishment of the nomogram

This study utilized the Cox regression model and the R package “rms” to develop a nomogram that predicts OS at 1-, 3-, and 5-year time frames. The accuracy of the nomogram was assessed using the C-index, while calibration plots were employed to visually compare the predicted and observed OS at 1-, 3-, and 5-year intervals.

### Statistical analysis

All statistical analyses were performed using R version 4.1.3 (https://www.r-project.org/) and its adequate packages. Statistical significance was set at p ≤ 0.05.

### Data availability

The source of datasets supporting the conclusions are included in this article.

## RESULTS

### Interrogating the cellular constitution of ovarian cancer at single-cell resolution

**Figure 1:**
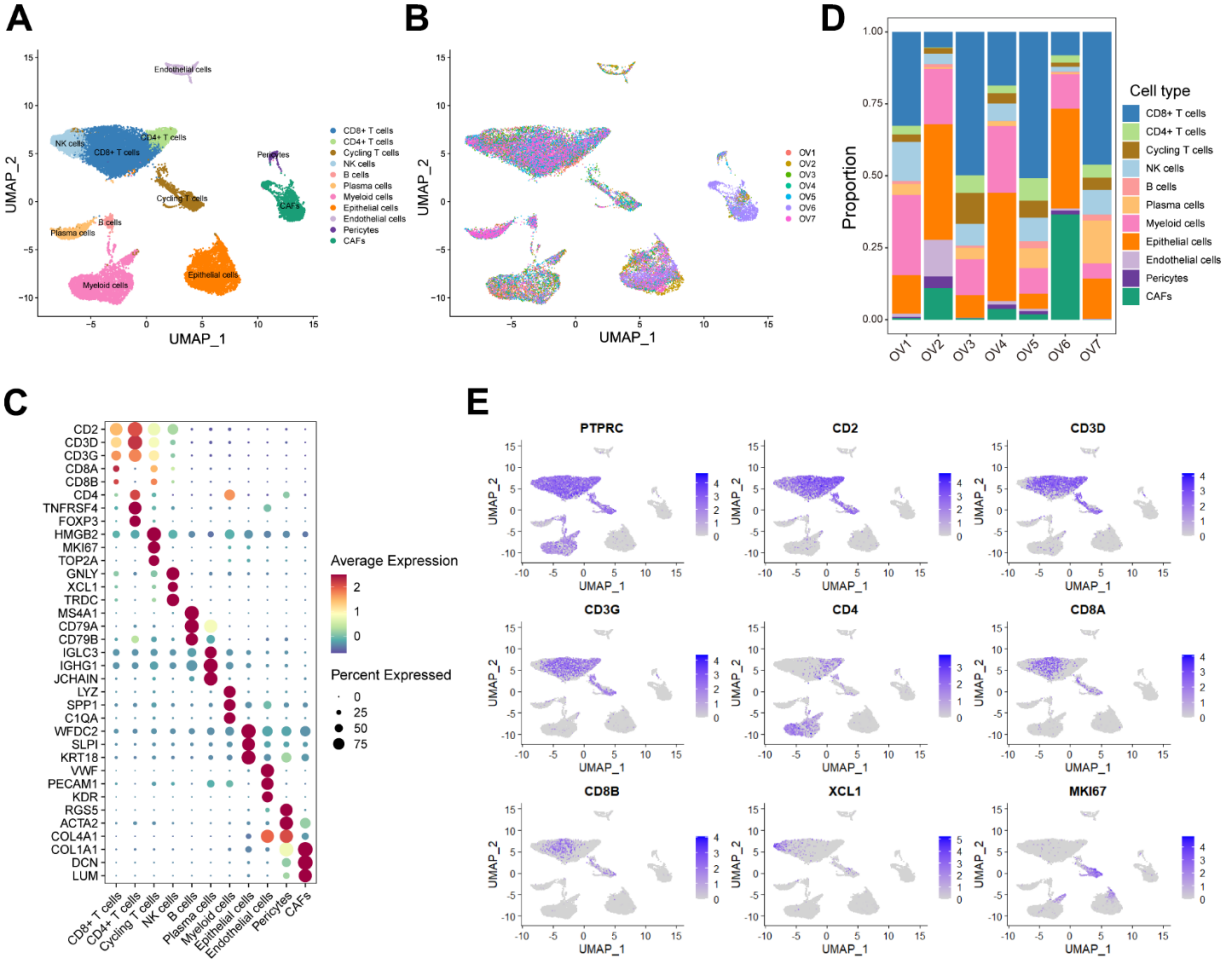
**Interrogating the cellular constitution of ovarian cancer at single-cell resolution.** (**A**, **B**) UMAP plot showing the major cell subpopulations in ovarian cancer. (**C**) Bubble heatmap showing expression levels of selected signature genes in ovarian cancer. Dot size indicates fraction of expressing cells, colored based on normalized expression levels. (**D**) Relative proportions of diverse cell types across each sample. (**E**) Feature plots to further identify various immune cells, based on the expression levels of marker genes.

In order to minutely investigate the cellular constitution of ovarian cancer at single-cell resolution and identify markers of CD8^+^ T cells, we re-analyzed the scRNA-seq data of tumors from seven treatment-naive ovarian cancer samples. Firstly, we integrated these data and corrected the potential batch effects using Harmony algorithm. After strict quality control and data filtering, data of 21785 genes within 27045 cells was obtained. We identified three major compartments within the TME of ovarian cancer, including the epithelial subset, immune subset and stromal subset (Figure 1A). UMAP visualization showed that seven scRNA-seq data were integrated and mixed uniformly (Figure 1B). By interrogating a list of classical lineage markers, we annotated the subpopulations among three main cellular subsets. For example, the stromal subset consisted of endothelial cells identified by expressions of *VWF*, *PECAM1* and *KDR*, pericytes with high *RGS5*, *ACTA2*, and *COL4A1* expressions, and cancer-associated fibroblasts (CAFs) identified by significant expressions of *COL1A1*, *DCN* and *LUM* (Figure 1C). Additionally, we identified several immune subpopulations including myeloid cells, NK cells, T cells, B cells, and plasma cells in the ovarian TME. Interestingly, a cycling T cell subpopulation which showed markers of both T cell lineage (*CD2*, *CD3D* and *CD3G*) and cell proliferation (*HMGB2*, *MKI67* and *TOP2A*) was identified. All major cell types were represented across all seven tumors, but showed diverse composition patterns (Figure 1D). For example, the CD8^+^ T cells were enriched in OV-1, OV-3, OV-5 and OV-7 samples, while the CAFs were mainly enriched in the OV-6 sample. These observed variations in cellular composition might partly explain the inter-tumoral heterogeneity. Considering the boundary of NK cells and T cells on the UMAP plot was equivocal, we devoted to minutely discriminate distinct cellular subsets. The NK cell subpopulation was then identified based on the negative expression of canonical T cell lineage markers including *CD2*, *CD3D*, *CD3G*, *CD4*, *CD8A* and *CD8B*, along with specifically high expression of the chemokine *XCL1*, as well as *GNLY* and *TRDC* (Figure 1C, 1E). Furthermore, CD4^+^ T cells and CD8^+^ T cells were distinguished based on the expression of *CD4*, *CD8A* and *CD8B* (Figure 1C–1E). Taken together, we minutely interrogated the cellular constitution of ovarian cancer at single-cell resolution and identified cellular markers of each subpopulation, such as the CD8^+^ T cell subpopulation.

### Construction and validation of a prognostic model based on specific CD8^+^ T cell markers in ovarian cancer

Conducting differential analyses on the scRNA-seq data, we calculated a list of 1020 genes as the markers of the CD8^+^ T cell subpopulation (Supplementary Table 1). As expected, the Gene Ontology (GO) enrichment analysis exhibited that these markers were enriched in multiple T cell-associated pathways, including T cell differentiation, T cell receptor signaling pathway and alpha-beta T cell activation (Supplementary Figure 1A). Moreover, the Kyoto Encyclopedia of Genes and Genomes (KEGG) enrichment analysis showed a similar result, including enrichment of signaling pathways such as T cell receptor signaling pathway, Th1 and Th2 cell differentiation, Th17 cell differentiation as well as PD-L1 expression and PD-1 checkpoint pathway in cancer (Supplementary Figure 1A).

**Figure 2:**
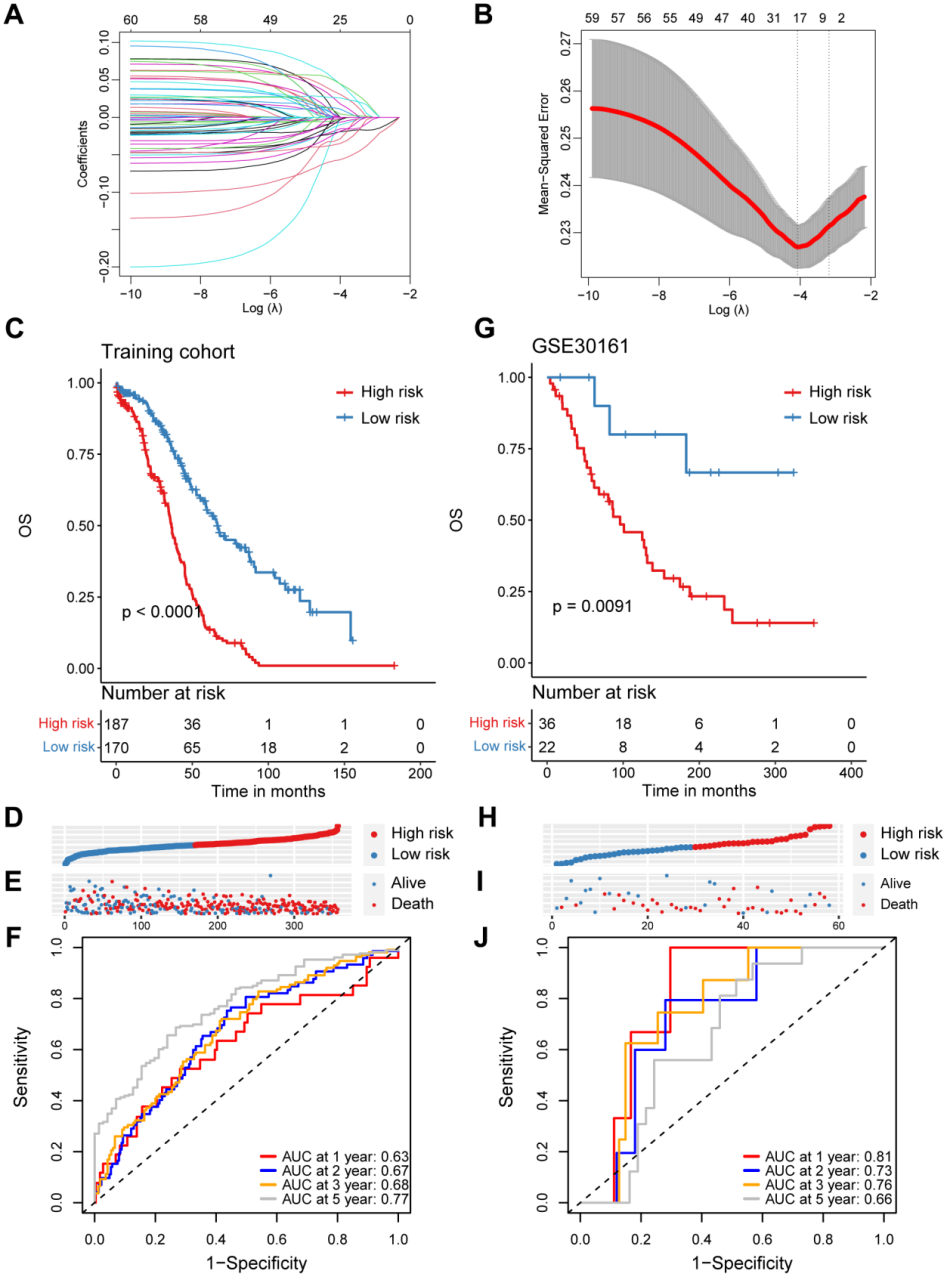
**Construction and validation of a prognostic model based on specific CD8^+^ T cell markers in ovarian cancer.** (**A**) Coefficient profiles in the LASSO regression model. (**B**) Cross-validation for tuning parameter selection in the LASSO regression. (**C**) Kaplan-Meier survival analysis was performed on the relationship between the risk score and OS using the TCGA training cohort. (**D**) The rank of risk score in the TCGA training cohort. (**E**) Survival status in the TCGA training cohort. (**F**) Time-dependent ROC curve analysis of the prognostic model (1, 2, 3, and 5 years) in the TCGA training cohort. (**G**) Kaplan-Meier survival analysis was performed on the relationship between the risk score and OS using the GSE30161 validation cohort. (**H**) The rank of risk score in the GSE30161 validation cohort. (**I**) Survival status in the GSE30161 validation cohort. (**J**) Time-dependent ROC curve analysis of the prognostic model (1, 2, 3, and 5 years) in the GSE30161 validation cohort.

In order to investigate the prognostic role of CD8^+^ T cells in ovarian cancer, we firstly carried out a univariate Cox regression analysis to identify potential prognostic markers associated with CD8^+^ T cells for ovarian cancer in the TCGA cohort. A total of 64 genes was found to be correlated with the ovarian cancer prognosis (Supplementary Table 2). Subsequently, we performed the LASSO regression analysis to reduce the number of prognostic genes and finally identified 17 prognostic signature genes, including *SLFN5*, *CD40LG*, *EMB*, *ISG20*, *CD226*, *CCR7*, *S1PR4*, *RNF167*, *MLLT3*, *PLEKHF1*, *RNPEPL1*, *STK39*, *FXYD5*, *BTN3A3*, *WNK1* and *SUSD3* (Figure 2A, 2B). Based on the coefficients and expression levels of these prognostic signature genes, we computed the risk score for each sample in the TCGA ovarian cancer cohort, which served as our training set. Subsequently, we categorized the ovarian cancer patients in the TCGA training cohort into high-risk and low-risk groups using the median risk score as the threshold. Our analysis revealed that patients in the high-risk group exhibited markedly inferior outcomes (Figure 2C–2E). To assess the effectiveness of our CD8^+^ T cell-associated risk model, we presented receiver operator characteristic (ROC) curves and determined the area under the ROC curve (AUC) values at 1, 2, 3, and 5 years, which were 0.63, 0.67, 0.68, and 0.77, respectively (Figure 2F).

To validate the reliability of the CD8^+^ T cell-related prognostic signature, we conducted additional validation tests in several independent datasets. Similarly, in the GSE30161 cohort, we stratified ovarian cancer patients based on the same risk score, consistent with the approach used in the TCGA training set and observed that patients in the high-risk group had poorer prognoses (Figure 2G–2I). The AUC values for the risk score in the GSE30161 dataset were 0.81 for 1 year, 0.73 for 2 years, 0.76 for 3 years, and 0.66 for 5 years (Figure 2J). Additionally, ovarian cancer patients with higher risk score in the GSE63885 dataset also exhibited significantly inferior prognoses (Supplementary Figure 2A–2C). The AUC values for the CD8^+^ T cell-related risk score in the GSE63885 dataset were 0.81 for 1 year, 0.73 for 2 years, 0.76 for 3 years, and 0.66 for 5 years (Supplementary Figure 2D). In summary, we have developed and validated a novel CD8^+^ T cell-related prognostic signature for predicting ovarian cancer outcomes.

### Functional and genomic features of high/low-risk groups

**Figure 3:**
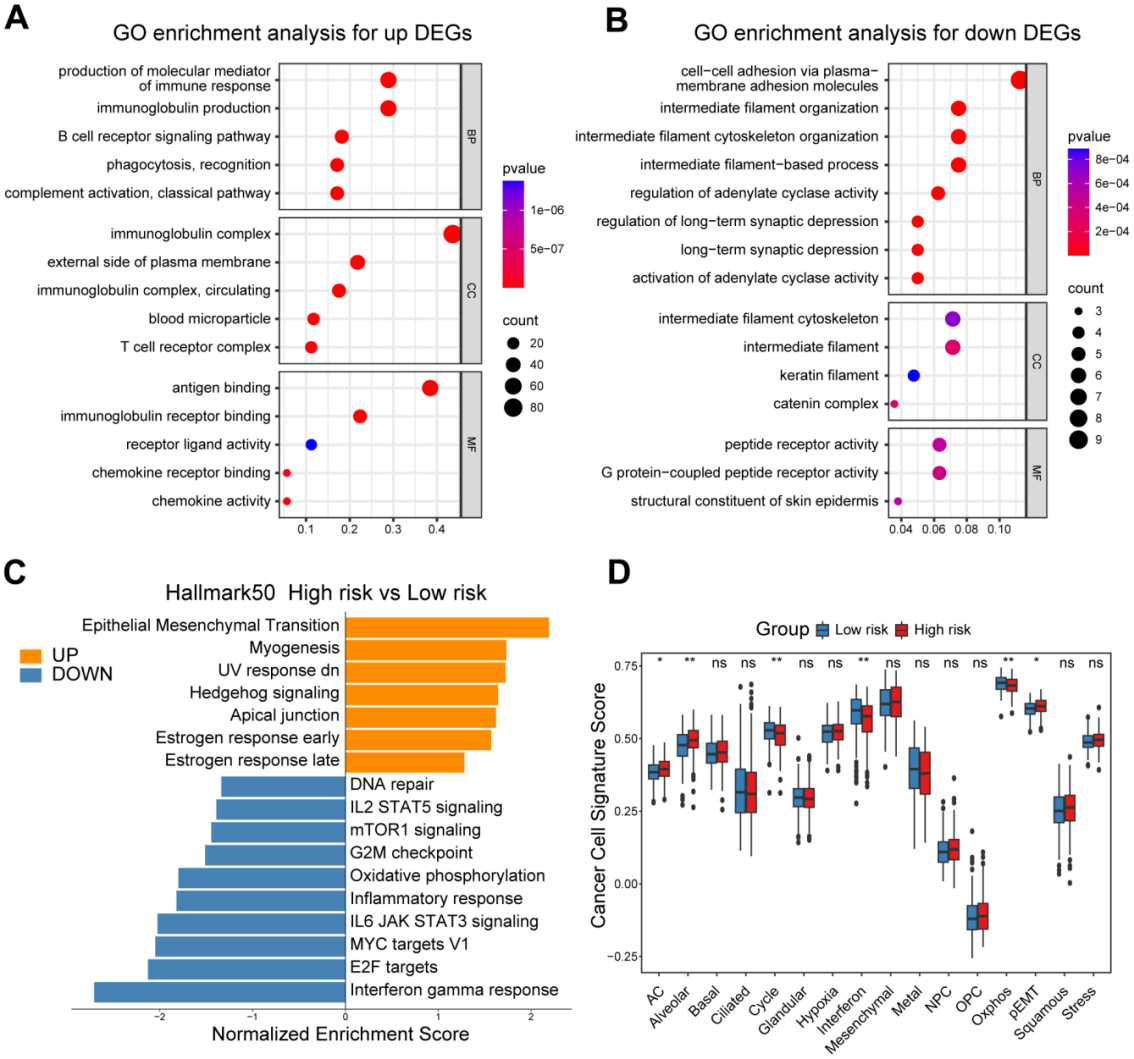
**Functional and genomic features of high/low-risk group patients.** (**A**, **B**) GO enrichment of DEGs in high/low-risk groups. (**C**) Bar plot showing different pathways enriched in high/low-risk groups of ovarian cancer calculated by GSEA. (**D**) Boxplots showing the signature score of 16 cancer cell states in high/low-risk groups of ovarian cancer scored by GSVA. Paired two-sided Wilcoxon test. The asterisks represent the statistical P-value (*p<0.05; **p<0.01; ***p < .001; ****p < 0.0001; ns p>0.05).

In our quest to elucidate the underlying mechanisms explaining the prognostic significance of the CD8^+^ T cell-related risk score, we initially set out to explore its functional and genomic attributes. GO enrichment analysis revealed that the high-risk group patients showed significant enrichment in multiple immune-related pathways, such as the B cell receptor signaling pathway, T cell receptor complex and antigen binding pathways (Figure 3A). In contrast, the intermediate filament cytoskeleton organization, catenin complex and G protein-coupled peptide receptor activity pathways were found to be enriched in the low-risk group patients (Figure 3B). In addition, the results of GSEA indicated that patients in the high-risk group had higher enrichment scores in pathways associated with epithelial-mesenchymal transition (EMT), Hedgehog signaling, and estrogen response (Figure 3C and Supplementary Figure 3A–3C), while those in the low-risk group showed elevated enrichment scores in pathways related to interferon-gamma response, DNA repair, and MYC targets V1 (Figure 3C and Supplementary Figure 3D–3F). In order to obtain further insights into the transcriptional heterogeneity among ovarian cancers, we conducted the GSVA algorithm to calculate 16 recurrent cancer cell states, which interacting with the TME to take shape organized systems qualified to promote immune escape, metastasis and drug resistance [18, 21]. We found the high-risk group patients harbored higher signature scores of astrocyte (AC)-like, alveolar and partial epithelial-mesenchymal transition (pEMT) modules (Figure 3D). Nevertheless, the cell cycle, interferon and oxidative phosphorylation modules were enriched among low-risk group ovarian cancer patients (Figure 3D).

Moreover, we illustrated and compared the mutation profiles of ovarian cancer patients in the high-risk and low-risk groups (Supplementary Figure 4A). Surprisingly, we found that TMB scores, which have vital potential to predict immunotherapy responses, were markedly elevated in the low-risk group patients, and negatively correlated with the CD8^+^ T cell-associated risk score (Supplementary Figure 4B–4C).

### Dissection of tumor immune microenvironment features based on CD8^+^ T cell-related prognostic signature

**Figure 4:**
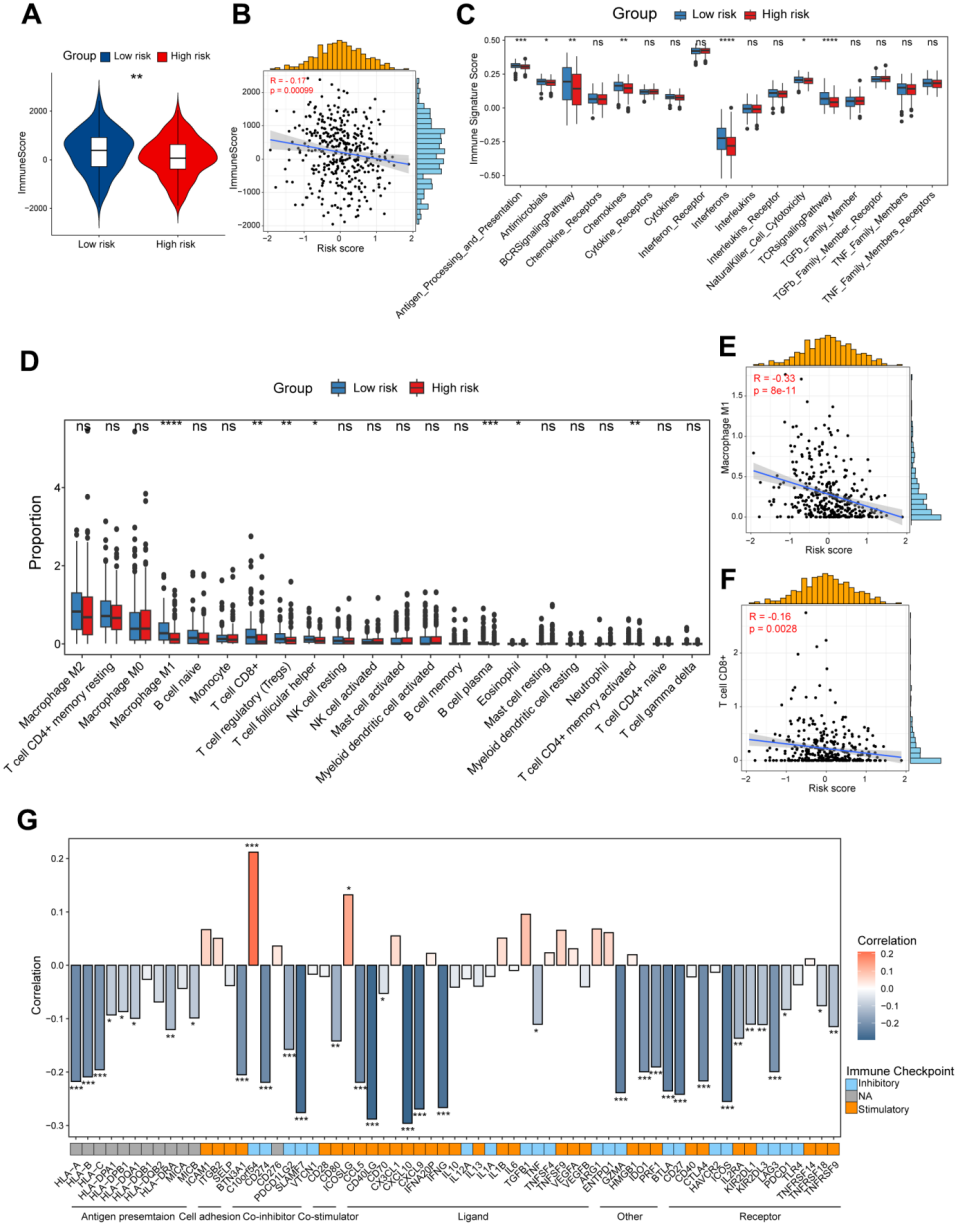
**Dissection of tumor immune microenvironment features based on CD8^+^ T cell-related prognostic signature.** (**A**) Immune score in high/low-risk groups. (**B**) Spearman correlation between Immune score and risk score. (**C**) Boxplots showing the signature score of 17 immune pathways in high/low-risk groups of ovarian cancer scored by GSVA. Paired two-sided Wilcoxon test. (**D**) Boxplots showing the proportion of 22 immune cells in high/low-risk groups of ovarian cancer estimated by CIBERSORT. Paired two-sided Wilcoxon test. (**E**, **F**) Scatter plots showing the correlation between the risk score and the proportion of M1-like macrophages and CD8^+^ T cells (**G**) Bar plot of the correlation between immunomodulators and the risk score. The asterisks represent the statistical P-value (*p<0.05; **p<0.01; ***p < .001; ****p < 0.0001; ns p>0.05).

To unveil the disparities in the immune milieu between the high-risk and low-risk group ovarian cancer patients, we first calculated the immune score using the ESTIMATE method. The results indicated that the immune score was dramatically higher in the low-risk group patients compared to high-risk patients, and it was negatively associated with this risk score (Figure 4A, 4B). Moreover, we interrogated a series of immune signature score in two risk group patients, and found that the low-risk group showed higher scores in signatures related to antigen processing and presentation, B cell receptor (BCR) signaling pathway, T cell receptor (TCR) signaling pathway, and interferons (Figure 4C). Subsequently, the CIBERSORT algorithm was performed to speculate the infiltration levels of diverse immune cell types in the ovarian cancer immune microenvironment. It was obviously indicated that high-risk patients had lower fractions of the M1-like macrophages, CD8^+^ T cells, regulatory T cells and follicular helper T cells (Figure 4D). In addition, the risk score showed a significantly correlation with the infiltration levels of the M1-like macrophages, CD8^+^ T cells (Figure 4E, 4F). Finally, we explored the potential association between our CD8^+^ T cell-related risk score and different immunomodulators. As shown in the bar plot, the risk score exhibited markedly positive correlation with *C10orf54* and *ICOSLG*, but significantly negative correlation with *CXCL10*, *CD40LG* and *IFNG* (Figure 4G). Taken together, these results indicated us the high/low-risk patients had distinct immune cell infiltration and immune features, which might potentially contribute to different outcomes of ovarian cancers.

### High/low-risk group patients differ in response to immunotherapy

**Figure 5:**
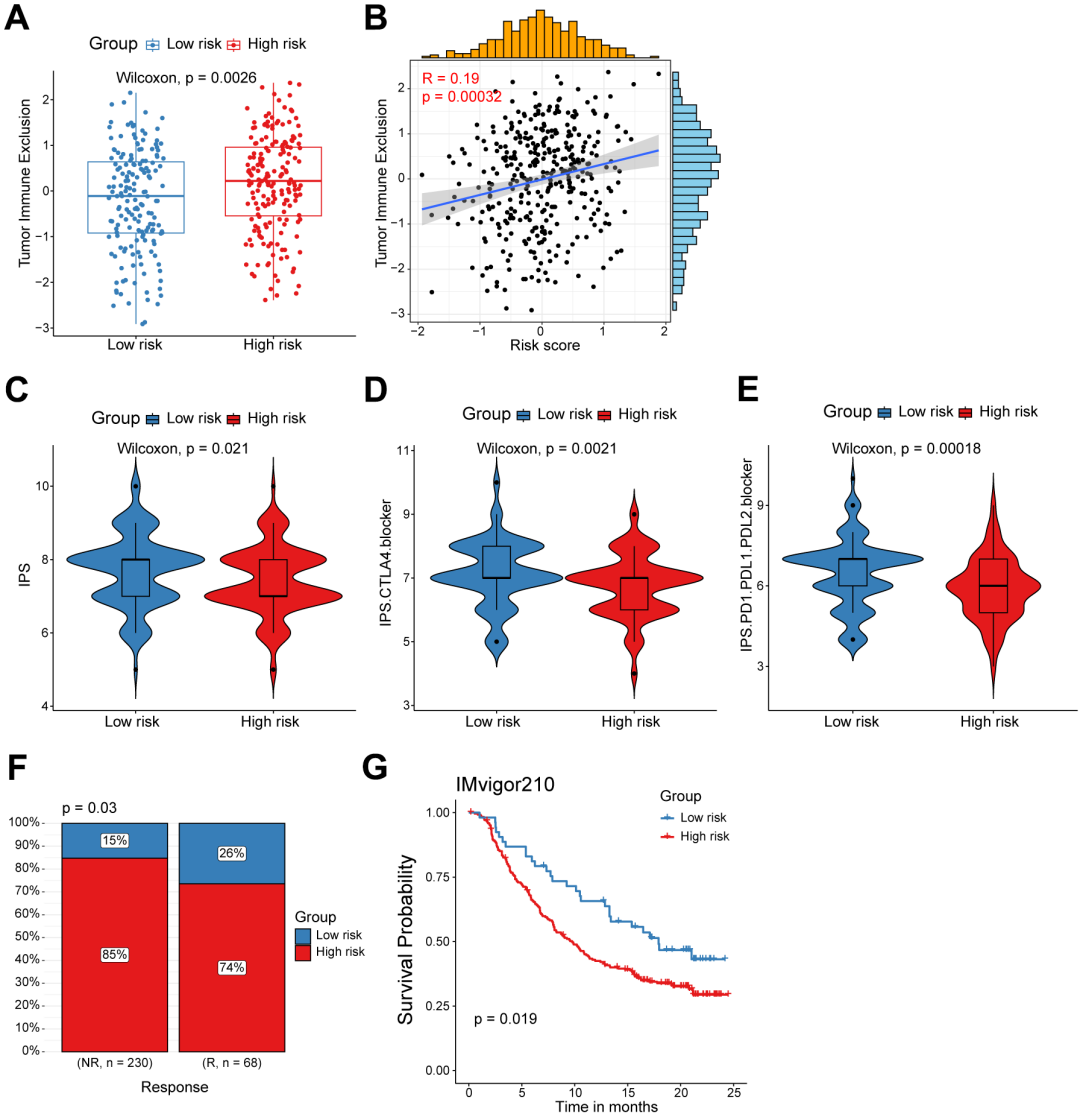
**High/low-risk group patients differ in response to immunotherapy.** (**A**) Tumor Immune Exclusion score in high/low-risk groups. (**B**) Spearman correlation between Tumor Immune Exclusion score and risk score. (**C**–**E**) Immunophenoscore (IPS) in high/low-risk groups. (**F**) Proportion of immunotherapy responses between high/low-risk groups. (**G**) Kaplan-Meier survival analysis was performed on the relationship between the risk score and OS in the IMvigor210 immunotherapy cohort.

Considering the evident correlation between the CD8^+^ T cell-associated risk score and diverse immune checkpoints, we subsequently devoted to figuring out whether the risk score correlated with the tumor immune exclusion score, which was recognized as one of immunotherapy predictors [22]. As expected, we found that ovarian cancer patients in the high-risk group harbored a higher tumor immune exclusion score, which positively associated with the risk score (Figure 5A, 5B). To evaluate the response of ovarian cancers in different risk groups to immune checkpoint inhibitors (ICIs), we calculated the IPS score (including CTLA4 blocker and PD-1/PD-L1/PD-L2 blocker) was much significantly higher in the low-risk group patients (Figure 5C–5E). This suggested that ICIs targeting CTLA4 or PD-1/PD-L1/PD-L2 are more suitable for low-risk patients rather than the high-risk group patients. Finally, to further validate the value of our CD8^+^ T cell-related prognostic model in predicting the immunotherapy response, we analyzed RNA-seq data of pretreatment samples of 298 bladder cancer patients from the IMvigor210 cohort before anti-PD-L1 treatment. We found high-risk patients were mainly in the non-response group, and patients with higher risk score also exhibited inferior outcomes as showed in the Kaplan-Meier analysis (Figure 5F, 5G). Collectively, the above findings indicate that patients in the low risk-group are more likely to benefit from immunotherapy, and the CD8^+^ T cell-related risk score could act as a potential biomarker to distinguish ovarian cancer patients who may benefit from immunotherapy.

### Distinct drug sensitivities were observed among high/low-risk group patients

**Figure 6:**
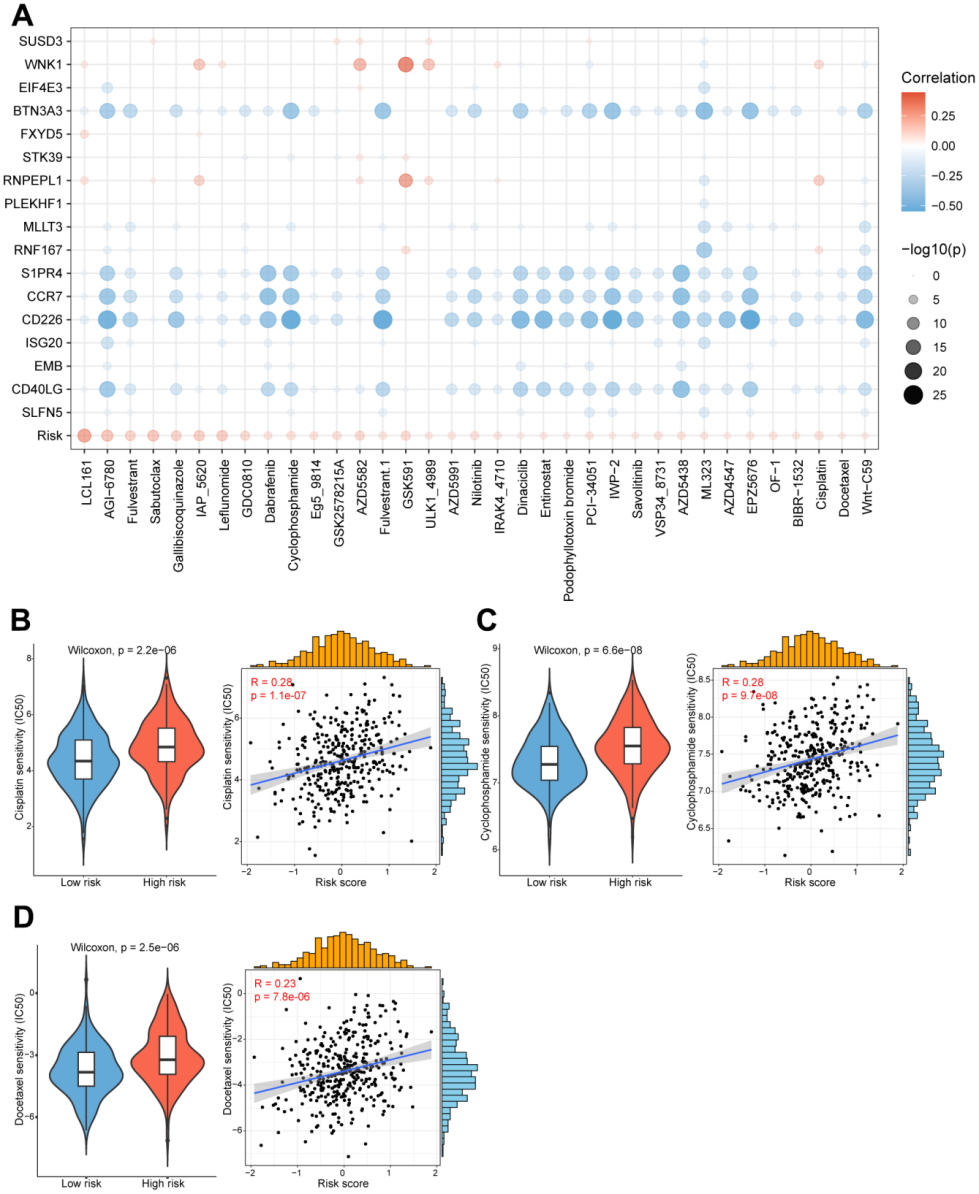
**High/low-risk group patients differ in drug sensitivity and response to immunotherapy.** (**A**) Bubble plot showing the relationship between drugs, risk score, and model genes. (**B**) Violin plot showing the comparison of IC50 of cisplatin between high/low-risk groups, and scatter plot showing the correlation between the IC50 of drugs and the risk score. (**C**) Violin plot showing the comparison of IC50 of cyclophosphamide between high/low-risk groups, and scatter plot showing the correlation between the IC50 of drugs and the risk score. (**D**) Violin plot showing the comparison of IC50 of docetaxel between high/low-risk groups, and scatter plot showing the correlation between the IC50 of drugs and the risk score.

The substantial implication of tumor heterogeneity lies in the fact that distinct groups of patients exhibit varying responses to anti-cancer drug treatments, thereby exacerbating the risk of treatment failure and recurrence. Therefore, we determined to explore the potential relationship between our prognostic model and diverse anti-cancer drug sensitivity. The IC50 values of multiple drugs in ovarian cancer patients were calculated and compared between the two risk groups as shown in the bubble plot (Figure 6A). Interestingly, the results suggested that the IC50 values of cisplatin, cyclophosphamide and docetaxel were significantly higher in the high-risk patients, and positively associated with the CD8^+^ T cell-related risk score (Figure 6B–6D). It indicated that ovarian cancer patients in the high-risk group might be more resistant to standard chemotherapy regimens. Additionally, we also assessed the correlation between the model genes and these anti-cancer drugs. Specifically, the expression levels of *WNK1* and *RNPEPL1* were both conspicuously positively related to the IC50 of GSK591. On the other hand, the IC50 values of cyclophosphamide negatively correlated with the expression levels of *BTN3A3*, *S1PR4*, *CCR7*, *CD226* and *CD40LG* (Figure 6A). Summarily, our findings suggest that this novel CD8^+^ T cell-related prognostic model can be a reliable predictor for screening efficient drugs in ovarian cancers.

### Construction of a nomogram to forecast survival for ovarian cancer

**Figure 7:**
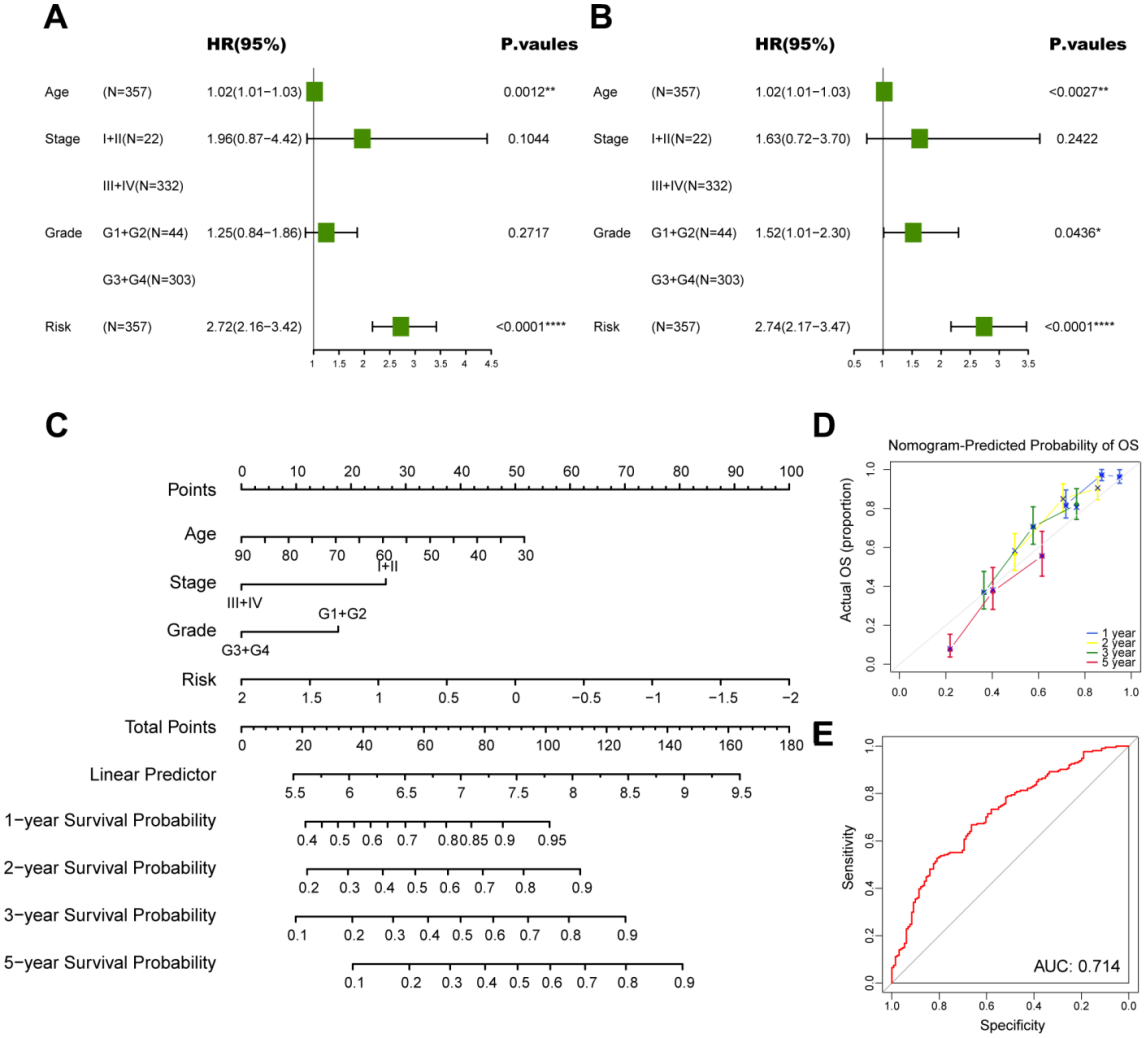
**Construction of a nomogram to forecast survival for ovarian cancer.** (**A**) Univariate analysis for the clinicopathologic characteristics and the risk score in TCGA cohort. (**B**) Multivariate analysis for the clinicopathologic characteristics and the risk score in TCGA cohort. (**C**) A nomogram was established to predict the prognostic of ovarian cancer patients. (**D**) Calibration analysis of the nomogram. (**E**) ROC curve analysis of the nomogram. The asterisks represent the statistical P-value (*p<0.05; **p<0.01; ***p < .001; ****p < 0.0001; ns p>0.05).

Subsequently, we conducted univariate and multivariate Cox analyses to determine whether the CD8^+^ T cell-associated risk score was a robust independent prognostic factor. The univariate Cox regression analysis revealed that the risk score was identified as a risk factor for ovarian cancer patients (Figure 7A). When adjusting for other confounding factors, the multivariate analysis also revealed that the risk score was still a prognostic factor independent of age, tumor stage and tumor grade (Figure 7B). Moreover, a predictive nomogram was established to improve the prognosis efficacy of our prognostic model and provide a quantitative and visual tool for estimating outcomes of ovarian cancer patients in the TCGA cohort (Figure 7C). As demonstrated by the calibration plots, the nomogram exhibited robust stability in quantifying the survival probabilities of ovarian cancer patients at 1, 2, 3, and 5 years (Figure 7D). These above results indicated that this constructed nomogram exhibited excellent prediction efficacy for ovarian cancer patients.

## DISCUSSION

Despite recent therapeutic advances, outcomes for patients with ovarian cancer still remain poor. Ovarian carcinoma is marked by a notable level of heterogeneity among patients, within individual patients, and within the tumor itself [23]. This complexity presents therapeutic challenges since the disease cannot be viewed as a singular entity. Therefore, the identification and development of novel biomarkers to contribute to patient-specific therapies and prolong survival are thirstily important and urgent. The TME of ovarian carcinoma is intricate and distinctive, and therapeutic strategies have aimed to address this heterogeneity by identifying the cellular components and comprehending their functions in tumor growth and metastasis. Aside from carcinoma cells, the TME in both fluid (ascites) and solid (omentum) niches includes immune cells, adipocytes and mesenchymal cells, while in the omentum, it also involves endothelial cells and pericytes [24]. In the immune cell compartment, CD8^+^ T cells serve essential functions in combatting intracellular pathogens and eradicating malignant cells in cancer. Nevertheless, the potential prognostic role of CD8^+^ T cell markers in ovarian cancer remains insufficient explored.

In contrast to bulk RNA-seq, which primarily assesses the average gene expression across all cells, scRNA-seq technologies have revolutionized molecular biology by providing the capability to measure transcriptome profiles at an unprecedented scale and resolution [25]. The integration of scRNA-seq and bulk RNA-seq analysis could provide substantial assistance in uncovering additional latent biological insights and represent the prevailing trend in current omics analysis and a future development direction. Here, we first minutely explored the CD8^+^ T cell markers of ovarian cancer by comprehensively analyzing a publicly obtained scRNA-seq dataset from ovarian cancer patients [9]. Based on canonical markers, we annotated plenty of cell subpopulations in the ovarian cancer TME. Primarily, epithelial cells were identified considering the expression level of *KRT18*, *SLPI* and *WFDC2*. Next, the stromal subset was further categorized into endothelial cells, pericytes and CAFs. For the immune compartment, myeloid cells, B and plasmas cells, NK cells and diverse T cell subpopulations were identified by classical markers. Subsequently, a novel CD8^+^ T cell-associated prognostic model was developed by univariate Cox regression and LASSO regression analyses for ovarian cancer patients in TCGA cohort, and further validated in other independent cohorts from the GEO dataset. We found high-risk group ovarian cancer patients were linked with significantly inferior outcomes. Importantly, patients with lower risk score exhibited dramatically elevated infiltration of immune cells, including M1-like macrophages and CD8^+^ T cells. In addition, we observed that patients in low-risk group exhibited a remarkably higher response rate to immunotherapy compared to those with high-risk score, suggesting that immune checkpoint blockade therapy is better suited for low-risk ovarian cancer patients. Besides, we screened a variety of drugs for high/low-risk ovarian cancer patients, and identified cisplatin, cyclophosphamide and docetaxel were more appropriate for patients in low-risk group. Of particular significance, we have developed an effective prognostic signature based on CD8^+^ T cell-associated genes and offered a novel and precise classification system as well as a therapeutic strategy for ovarian cancer patients.

Eventually, 17 CD8^+^ T cell-related genes, including *SLFN5*, *CD40LG*, *EMB*, *ISG20*, *CD226*, *CCR7*, *S1PR4*, *RNF167*, *MLLT3*, *PLEKHF1*, *RNPEPL1*, *STK39*, *FXYD5*, *BTN3A3*, *WNK1* and *SUSD3*, were herein screened to establish the novel prognostic model. Above genes have been partly found to be linked with CD8^+^ T cells in ovarian cancer and other cancers. For example, it has been reported that CD40L (encoded by *CD40LG*) has a significant impact on augmenting the population of CD8^+^ T cells by acting through CD40 receptors expressed on activated CD8^+^ T cells, and further exerts an influence on the generation of memory CD8^+^ T cells [26]. Moreover, CD226^high^ CD8^+^ T cell subpopulation in liver metastases was found to potentially determine the outcome of colorectal cancers undergoing chemotherapy and radical surgery [27]. A recent study reported that ablation of the immune cell-specific G protein-coupled receptor S1PR4 induced CD8^+^ T cell expansion to inhibit tumor growth and enhance chemotherapy efficacy [28].

Considering the high heterogeneity of ovarian tumors, we first attempted to divided patients into high/low-risk based on the median value of risk score. By conducting enrichment analyses, we found that low-risk group patients showed higher enrichment scores in the B cell receptor signaling pathway, classical complement activation and phagocytosis pathways. Consistent with this, ovarian cancer patients with a lower risk score had a significant elevated immune score calculated by ESTIMATE algorithm. Moreover, we found the infiltration of M2-like macrophages showed a comparable level among high/low-risk group patients. However, a lower infiltration level of M1-like macrophages was found in patients with a higher risk score. The destiny of macrophages depends on the surrounding conditions, which determine whether they adopt an inflammatory M1 response or an immune boosting M2 response. M1-polarized macrophages are stimulated by substances or inflammatory molecules while M2-polarized macrophages are triggered by interleukins, such as IL-4 and IL-13. The M1-like macrophages play a role in fighting, against bacteria and tumors whereas the M2-like macrophages primarily contribute to blood vessel formation, wound healing and immune regulation [29, 30]. Therefore, the M1-like macrophages might play a vital role in the improved prognosis among low-risk group patients. Additionally, the high-risk group patients also revealed a decreased infiltration level of CD8^+^ T cells, hinting weaker antitumor capacity, which could partly explain the inferior outcome of patients with higher risk score. Apart from revealing the heterogeneity of ovarian carcinoma in diverse aspects, we next devoted to developing a treatment landscape for ovarian cancer patients. Immunotherapy with ICIs has emerged as one of the pillars of cancer treatment, together with surgery, chemotherapy, and radiotherapy. Nevertheless, most patients fail to response to immunotherapies due to primary or acquired resistance. Inspired by this, we evaluated the response rate of immunotherapies between high/low-risk group patients and found that immune checkpoint blockades were more effective for patients with lower risk scores. Additionally, the sensitivity of a variety of drugs was interrogated among ovarian cancer patients. In order to enhance the clinical applicability of our model, we have comprehensively considered various clinical factors currently under consideration in the treatment of ovarian cancer. By integrating tumor staging, grading, and other pertinent factors, we have developed a forest plot capable of predicting multi-year survival rates for ovarian cancer patients. Through the analysis of chemotherapy regimens, PD-L1 immunotherapy, and the forest plot, clinicians can derive specific guidance for personalized treatment strategies tailored to ovarian cancer patients. In short, our research partly characterized the heterogeneity of ovarian cancers, and developed a treatment landscape including multiple effective therapeutic strategies for different ovarian carcinoma subpopulations.

In fact, while our prognostic model based on CD8^+^ T cells in ovarian cancer demonstrated outstanding performance in both the training and validation cohorts, there are still some limitations to consider. At first, the expression and prognostic role of the candidate CD8^+^ T cell marker genes at protein-level require further validation. Next, although we screened these prognostic genes based on CD8^+^ T cell markers, they were not solely expressed in CD8^+^ T cells. Finally, it is also worth noting that there might be some inherent biases to a certain extent due to the retrospective recruitment of ovarian cancer patients.

## CONCLUSIONS

In conclusion, we constructed and validated a novel prognostic signature based on CD8^+^ T cell markers identified by scRNA-seq analyses in ovarian cancer. The developed CD8^+^ T cell-related prognostic model in this research acts as an excellent predictor for clinical outcomes and therapeutic strategy choices for ovarian cancer patients. Besides, our study provides us a novel research direction of tumor-infiltrating immune cells in ovarian carcinoma.

## Supplementary Material

Supplementary Figures

Supplementary Table 1

Supplementary Table 2
