# Supplementary material for: Comprehensive single-cell and bulk RNA-seq analyses reveal a novel CD8+ T cell-associated prognostic signature in ovarian cancer
Source: Aging (Albany NY). 2024 Jun 25;16(12):10636–56. doi: 10.18632/aging.205966 (PMC11236322; doi:10.18632/aging.205966)
Supplement: Supplementary Figures [file aging-16-205966-s001.pdf]

SUPPLEMENTARY FIGURES

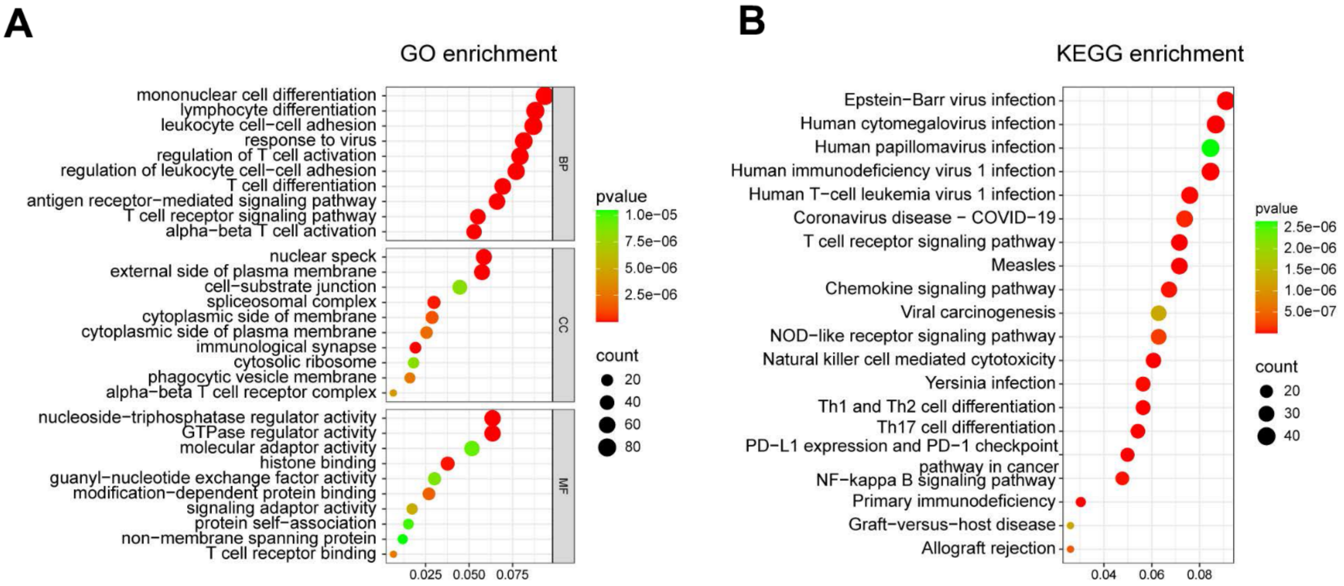

Supplementary Figure 1. GO/KEGG enrichment for CD8⁺ signature genes. (**A**) GO enrichment for CD8⁺ signature genes. (**B**) KEGG enrichment for CD8⁺ signature genes.

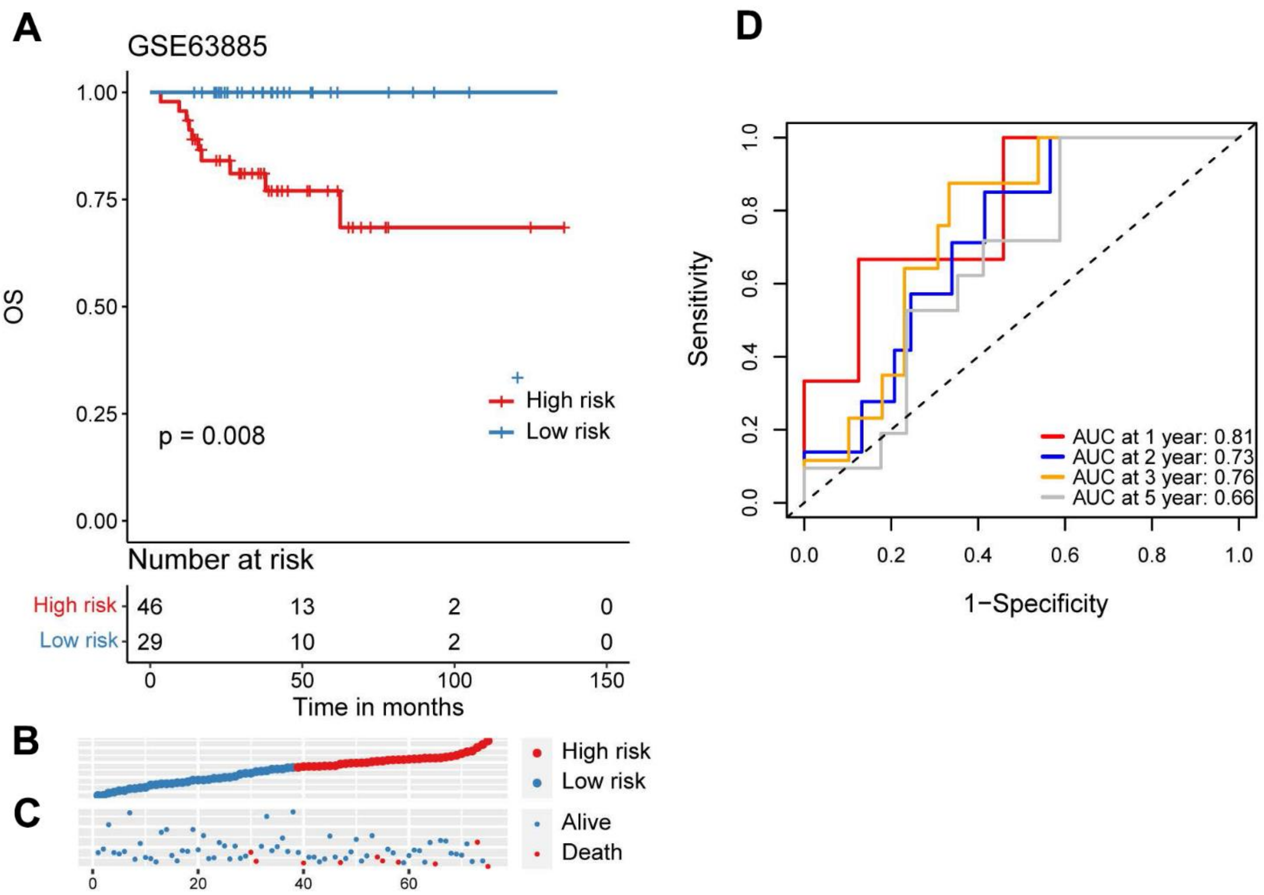

Supplementary Figure 2. Construction and validation of a prognostic model based on CD8⁺ T cell markers in ovarian cancer. (**A**) Kaplan-Meier survival analysis was performed on the relationship between the risk score and OS using the GSE63885 validation cohort. (**B**) The rank of risk scores in the GSE63885 validation cohort. (**C**) Survival status in the GSE63885 validation cohort. (**D**) Time-dependent ROC curve analysis of the prognostic model (1, 2, 3, and 5 years) in the GSE63885 validation cohort.

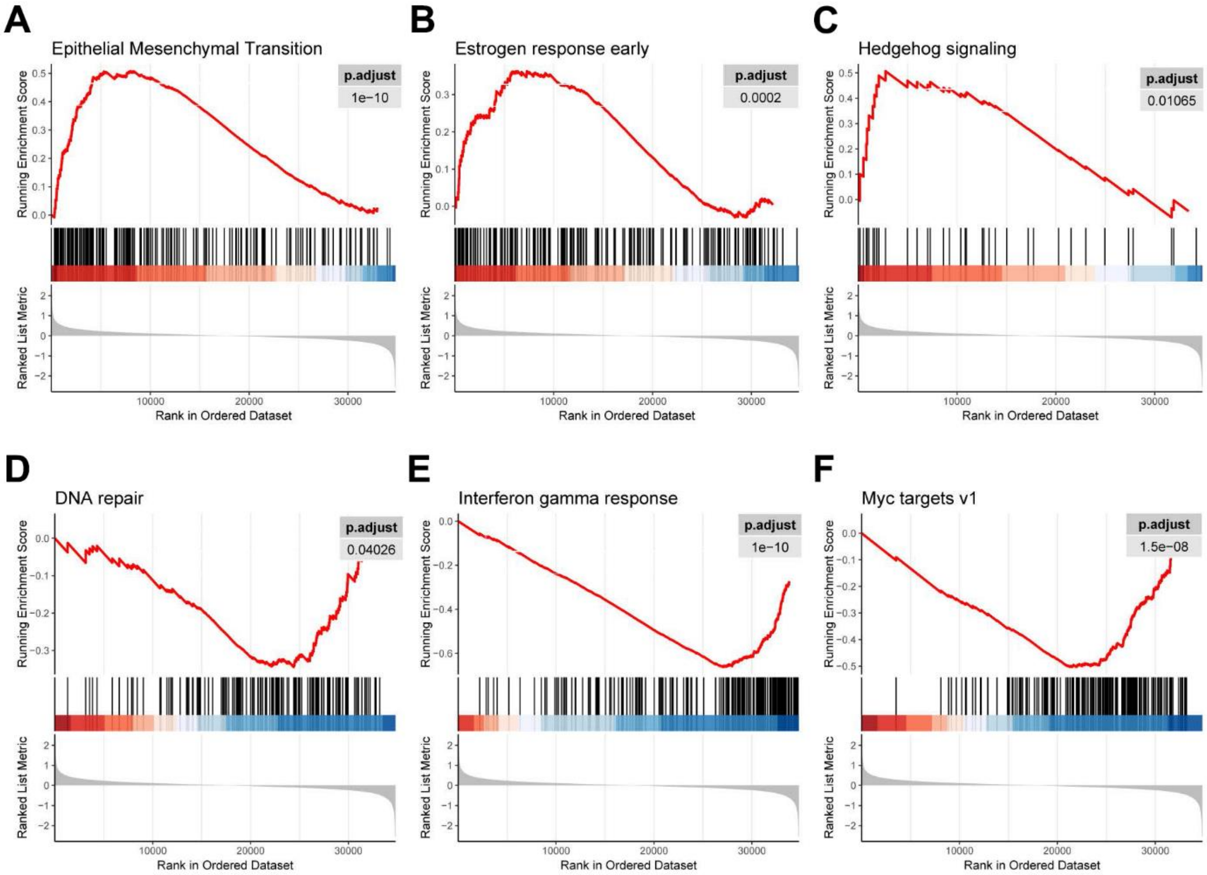

Supplementary Figure 3. Correlation between the risk score and pathway activities. (**A**–**C**) GSEA analysis showing the up-regulated pathways in the high-risk group. (**D**–**F**) GSEA analysis showing the up-regulated pathways in the low-risk group.

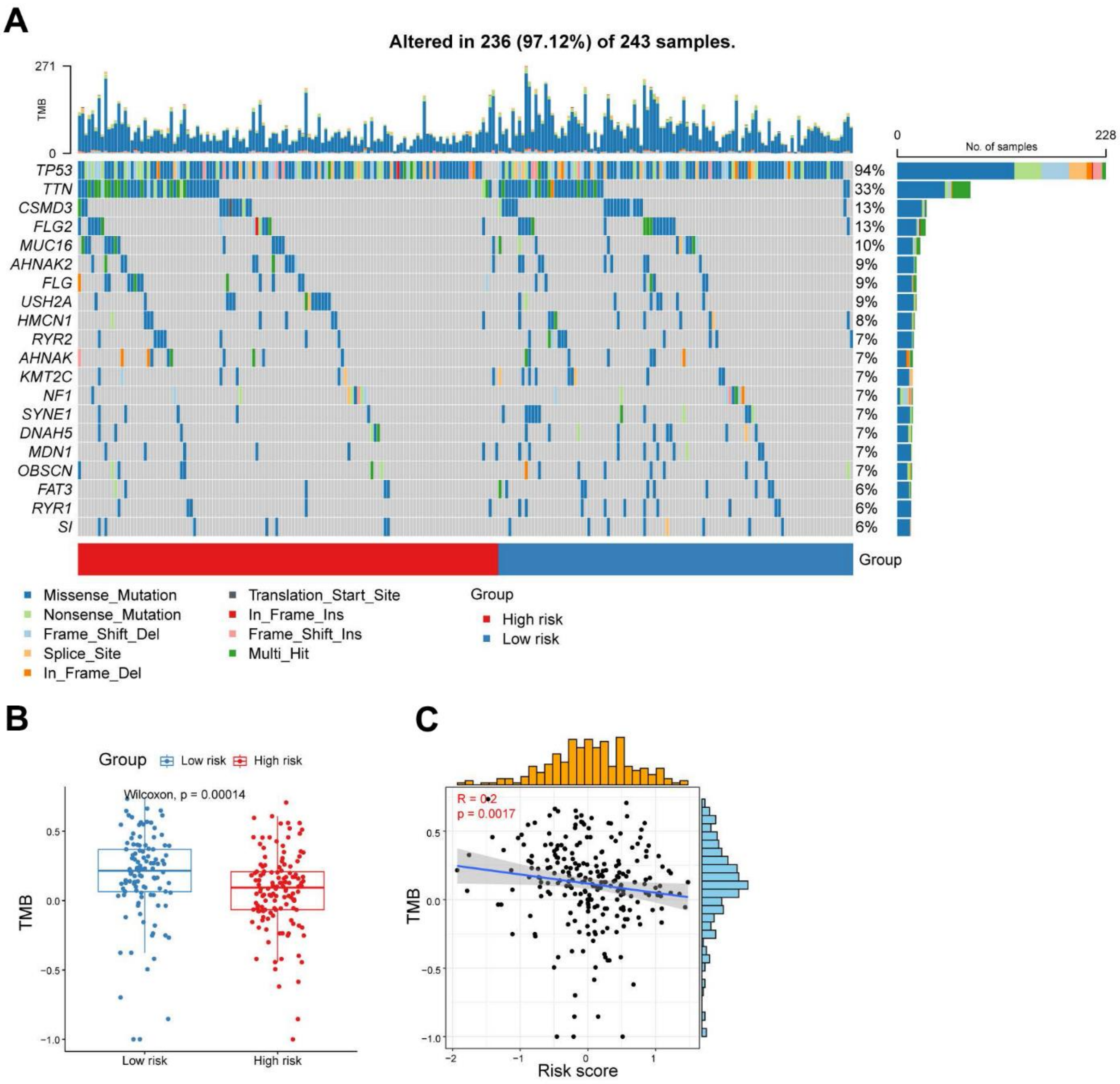

Supplementary Figure 4. Mutation analysis of high/low-risk groups (**A**). Comparison of the mutation landscape between groups with high/low risk. (**B**) TMB score in high/low-risk groups. (**C**) Spearman correlation between TMB score and risk score.
